# A randomised controlled trial of a complex intervention to reduce children’s exposure to secondhand smoke in the home

**DOI:** 10.1136/tobaccocontrol-2016-053279

**Published:** 2017-04-21

**Authors:** Elena Ratschen, Rebecca Thorley, Laura Jones, Magdalena Opazo Breton, Juliette Cook, Ann McNeill, John Britton, Tim Coleman, Sarah Lewis

**Affiliations:** 1 Department of Health Sciences, Mental Health and Addictions Research Group, University of York, York, UK; 2 Department of Epidemiology and Public Health, University of Nottingham, Nottingham, UK; 3 UK Centre for Tobacco and Alcohol Studies (UKCTAS), Nottingham, UK; 4 Public Health, Epidemiology & Biostatistics, Institute of Applied Health Research, University of Birmingham, Birmingham, UK; 5 National Addictions Centre, Institute of Psychiatry, King’s College London, London, UK; 6 Nottingham University Hospital NHS Trusts, Nottingham, UK; 7 Division of Primary Care, University of Nottingham, Nottingham, UK

**Keywords:** Secondhand smoke, Smoking Caused Disease, Addiction

## Abstract

**Objectives:**

Exposing children to secondhand tobacco smoke (SHS) causes significant harm and occurs predominantly through smoking by caregivers in the family home. We report a trial of a complex intervention designed to reduce secondhand smoke exposure of children whose primary caregiver feels unable or unwilling to quit smoking.

**Design:**

An open-label, parallel, randomised controlled trial.

**Setting:**

Deprived communities in Nottingham City and County, England

**Participants:**

Caregivers resident in Nottingham City and County in England who were at least 18 years old, the main caregiver of a child aged under 5 years living in their household, and reported that they were smoking tobacco inside their home.

**Interventions:**

We compared a complex intervention combining personalised feedback on home air quality, behavioural support and nicotine replacement therapy for temporary abstinence with usual care.

**Main outcomes:**

The primary outcome was change in air quality in the home, measured as average 16–24  hours levels of particulate matter of  < 2.5  µm diameter (PM_2.5_), between baseline and 12 weeks. Secondary outcomes included changes in maximum PM_2.5_, proportion of time PM_2.5_ exceeded WHO recommended levels of maximum exposure of 25  µg/mg^3^, child salivary cotinine, caregivers’ cigarette consumption, nicotine dependence, determination to stop smoking, quit attempts and quitting altogether during the intervention.

**Results:**

Arithmetic mean PM_2.5_ decreased significantly more (by 35.2 %; 95%  CI 12.7% to 51.9 %) in intervention than in usual care households, as did the proportion of time PM_2.5_ exceeded 25  µg/mg^3^, child salivary cotinine concentrations, caregivers’ cigarette consumption in the home, nicotine dependence, determination to quit and likelihood of having made a quit attempt.

**Conclusions:**

By reducing exposure to SHS in the homes of children who live with smokers unable or unwilling to quit, this intervention offers huge potential to reduce children’s’ tobacco-related harm.

**Trial registration number:**

ISRCTN81701383.

This trial was funded by the UK National Institute for Health Research (NIHR): RP-PG-0608-10020

## Introduction

The detrimental health effects of exposing children to secondhand tobacco smoke (SHS) are well established and include increased risks of lower respiratory infections,^1^ wheeze and asthma,^2^ middle ear disease,^3^ meningitis^4^ and sudden infant death syndrome.^5 6^ Most childhood SHS exposure occurs in the home environment,^5 6^ with 39% of English children who live with smokers exposed in the home on a regular basis.^7^ Notably, the highest levels of SHS exposure is found in the most socioeconomically disadvantaged families, where caregivers are more likely to be smokers and to smoke heavily,^8 9^ thus reinforcing the gradient of health inequalities through intergenerational perpetuation of tobacco dependence and harm.^10^

Although the optimal means of eliminating children’s exposure to SHS in the home is for caregivers to quit smoking, it is apparent that this cannot always be successfully or sustainably achieved.^11^ Where quitting smoking is not desired by or not achievable for caregivers, a ‘next best option’ to reduce children’s exposure is to support caregivers in making their homes completely smoke free.^11^ A range of approaches based on behaviour change theories and educational strategies has been proposed to help to protect children from exposure to SHS and to promote smoke-free homes (SFHs) for children.^12^ However, a review of interventions aimed at promoting SFHs,^13^ and a systematic review^14^ and separate meta-analysis^15^ of interventions aimed at reducing children’s exposure to SHS have found mixed evidence for effectiveness that was insufficient to recommend any one specific interventional strategy over another. A more recent systematic review^16^ and meta-analysis highlighted that measuring the effectiveness of SHS interventions can be problematic. It concluded that, although some studies that had used objective measures of SHS exposure had shown to be effective in reducing exposure to SHS in the home, air contamination through SHS was still overall significant following the intervention in the study homes. The authors further acknowledged that it is important that further effective interventions to promote SFHs are developed.^16^

On the basis of a UK feasibility study, which showed that providing families with personalised home air quality feedback combined with a motivational interviewing approach had potential to improve home air quality,^17^ and findings from our own development work,^18 19^ we designed a complex intervention comprising behavioural and pharmacological support including personalised indoor air quality feedback. We hypothesised that air quality and other markers of SHS exposure (eg, saliva cotinine) and smoking behaviour (eg, numbers of cigarettes smoked in the home per day) in homes receiving the complex intervention would be significantly decreased compared with homes that did not. We now report results from a randomised controlled trial assessing the effectiveness of this intervention in reducing children’s exposure to SHS in the home.

## Methods

### Trial design

We conducted an open-label, parallel, randomised controlled trial in deprived communities in Nottingham City and County in England. Ethical approval for the trial was granted by the National Research Ethics Service West Midlands, Solihull, in September 2012.

## Participants

We recruited and followed up caregivers resident in Nottingham City and County in England who were eligible to participate if they were at least 18 years old, were the main caregiver of a child aged under 5 years living in their household (in households with more than one child under 5 years, the youngest was defined as ‘index child’ for the study) and reported that they were smoking tobacco inside their home. Potential participants who were already trying to quit smoking or had attempted to do so in the 3 months preceding recruitment were excluded, as were pregnant smokers, those planning a pregnancy or breast feeding during the intervention period, those with health-based contraindications to nicotine replacement therapy (NRT) use and those living in hostels or refuges.

Participants were recruited from 81 English ‘Sure Start’ Children’s Centres across Nottinghamshire, which offer free services for disadvantaged families with children up to 5 years of age, designed to improve health, language and readiness to learn before school.^20^ Potential participants were identified and approached with the help of Children’s Centre managers and through attendance of ‘outreach’ activities such as play sessions. The research team also liaised with community-based Health Visitors (registered nurses who offer health advice and support to families with preschool children) in deprived areas. An invitation to take part was mailed from a large inner city general practice to all eligible caregivers. Participants received an inconvenience allowance of £50 high street shopping voucher on completion of data collection.

### Randomisation and masking

After obtaining written informed consent, caregivers (and their households) were randomised either to the complex intervention or to usual care in a 1:1 ratio based on a computer-generated pseudo-random code using random permuted blocks of randomly varying size created by the Nottingham Clinical Trials Unit. The trial necessarily used an open design, but analysis was carried out blind to treatment group.

## Procedures

### Data collection

In both treatment groups, data were collected during home visits at baseline, 7 and 12 weeks, carried out by a researcher and a smoke-free homes advisor (SFHA), trained according to the UK National Centre for Smoking Cessation and Training Level one standard. Participants completed questionnaires on socioeconomic status, their own and the index child’s health, family and household composition, current smoking behaviour and beliefs relating to home smoking with children Indoor air was sampled for up to 24 hours using a Sidepak Aerosol Monitor AM510 (TSI Instruments Ltd, High Wycombe, UK), positioned in the main living area. Monitors were set with the calibration factor of 0.30 by the manufacturer to measure particle sizes of secondhand smoke.^21^ PM_2.5_ data were logged at 60 s intervals throughout the data collection period, which varied between 16 and 24 hours, depending on when a collection of the monitor could be arranged with participants. Two air monitors were affected by calibration error, resulting in their calibration factor being set to 1.0 for a period of time throughout the study. Affected data were converted to the correct calibration level retrospectively using *Trackpro* software in a standard approach, and participants were notified. Saliva samples to measure cotinine, the major proximate metabolite of nicotine and a biological marker for SHS exposure^16 22^ were taken from the index study child during the home visit by a member of the research team in the presence of the caregiver, using appropriate swabs (*Salimetrics* infant swabs). Samples were analysed in a quantitative assay using liquid chromatography tandem mass spectrometry (ABS Laboratories).

### Intervention

The intervention comprised feedback on the air quality measured in the home; behavioural support on how to create an SFH delivered by a specialist SFHA; and NRT for temporary abstinence or for cutting down tobacco smoking in the home, provided at baseline, 7 and 12 weeks.

#### Air quality measurement and personalised feedback

Using *Trackpro* software installed on the air monitors, the research team converted data collected during the three periods of air quality measurement (baseline, week 7 and week 12) into a graphical format that could be easily explained to the participant immediately after each measurement period, relating the information to the WHO recommended 24 hours of PM_2.5_ concentrations below 25 µg/m^3^ per 24-hour period.^23^ Participants were shown the graphs with attention drawn to periods of time that showed particularly high or low SHS exposure in the home, supported by the discussion of reasons for high or low values and of strategies to reduce exposure both in general and during periods when levels were particularly high. Data collected at weeks 7 and 12 were super imposed on the baseline graph to enable the comparison of PM_2.5_ levels over the intervention period.

#### Behavioural support from specialist smoke-free home advisors

Behavioural support for the intervention group comprised face-to-face home visits by a specialist SFHA offered on four occasions (1–2 days after baseline measurements, at 3, 7 and 12 weeks) and lasting for up to 1 hour. The SFHA and participants discussed current smoking behaviours in and around the home using a ‘Smokefree Home Factsheet’ for reference (see online supplementary appendix 1), and explored personalised strategies to avoid smoking indoors in the context of individual circumstances, the availability of outdoor spaces and the need for constant supervision of children. In addition, participants received a minimum of two proactive phone calls (during the second week and after 5 weeks of follow-up) and the offer to contact the SFHA via phone or text message on an ad hoc basis for support during the intervention period during office hours, if required. Adult smokers living in the same household were eligible to receive the same behavioural and pharmacological support.

10.1136/tobaccocontrol-2016-053279.supp3 Supplementary file 1

#### Nicotine Replacement Therapy

Nicotine replacement therapy (NRT) products licensed for temporary abstinence and cutting down on smoking, including mouth spray, nicotine gum, lozenges, inhalers and patches, were offered to all participants free of charge and dispensed by the SFHA. Participants were provided with samples of small quantities of the NRT products to support an informed choice on which one (or which combination) may be best suited to them. Suitability and use were reviewed during the 1–2 weeks proactive phone call and at each home visit and a further 4 or 5 weeks of product dispensed at the 3-week and 7-week visits, if appropriate. Thus, a maximum of 12 weeks of free NRT was dispensed.

Participants who expressed an interest in quitting smoking altogether were advised that NRT products could be used for this purpose and received advice on a quitting strategy, or where interest was expressed at the 12-week appointment, a referral to the local specialist stop smoking services.

### Usual care

Participants randomised to the usual care group were provided with a ‘Smoke Free Homes resource pack’, developed by the local Stop Smoking Service (SSS) as standard support routinely offered to caregivers with young children who smoked at the time the study was designed. The pack contained a fact sheet, booklet, door hangers, magnets, stickers, information on constituents of tobacco smoke and how to keep the family and home safe, and local stop smoking service contact information (see online supplementary appendix 1). On completion of the study at week 12, all participants in the usual care group received graphical feedback on their home air quality data and basic behavioural advice supported by the offer of 1 month’s supply of free NRT products for temporary abstinence and/or cutting down dispensed by the SFHA. During the course of the study, the ‘usual care’ resource pack offered by the local SSS was discontinued as standard care. As per protocol, it was, however, continued to be used for the purpose of this study.

## Outcomes

The primary outcome was the change in average home air quality (indoor 16–24 hour average PM_2.5_) between the baseline and 12-week measurement, compared between treatment groups, based on the rationale that, in contrast to other measures (such as saliva cotinine), this measure relates specifically to the intervention rather than secondhand smoke exposure in more general terms. Secondary outcome measures, also compared between treatment groups, included maximum concentrations of PM_2.5_, the proportion of time PM_2.5_ concentrations exceeded WHO recommended levels of maximum exposure of 25 µg/m^3^ per 24 hours period,^23^ salivary cotinine levels in the index child, caregiver-reported cigarette consumption in the home, caregivers’ Heaviness of Smoking Index (HSI),^24^; determination to quit, quit attempts and quitting altogether.

## Statistical analysis

An intention-to-treat analysis was performed using all randomised participants. Data on air quality were considered sufficient for analysis where 16 hours or more of particulate matter measurements had been obtained. The sample size was defined as 100 participating households per group, powered at 83% assuming alpha 0.05 and calculated to detect a 33% reduction between baseline and week 12 in log-transformed mean home air quality (PM_2.5_) in the intervention group compared with the usual care group.^16^

The primary outcome was compared between intervention and control groups using multiple linear regression, adjusted for baseline (basic model), and then adjusted for season, partner’s smoking status and multiple deprivation index as prognostic variables (adjusted model). Our primary analysis used a multiple imputation model, assuming that those participants who refused consent for follow-up were missing at random and included all relevant variables to predict average PM_2.5_ in the model (HSI, number of cigarettes smoked daily inside the house, demographic characteristics and the outcome measure). We carried out a range of sensitivity analyses using alternative assumptions about the missing data (complete case and last observation carried forward), using specific models for data over repeated measures (mixed model), excluding those participants with affected air quality readings due to calibration error, and using alternative definitions for our primary outcome variable. Alternative assumptions about missing data included a complete case model that assumes missing completely at random and a last observation carried forward model, assuming those lost to follow-up would have preserved similar air quality levels from the last observation recorded. Second, to account for repeated measures from the same units, we used a mixed methods approach incorporating all three repeated measurements with a random effect to allow for clustering by household. Third, to explore the impact of calibration factor errors, we performed the primary analysis excluding those participants affected by incorrect air quality readings and, finally, we used two alternative computations of mean PM_2.5_ including only those participants with paired measurements at baseline and week 12 obtained at exactly the same time of day for the household and across all households. For secondary outcomes, a complete case approach was used, with linear regression for continuous variables—log-transformed when appropriate—and logistic regression for categorical variables.

## Participant involvement

A lay representative was a coapplicant on the original research proposal and was involved in the early stages of study design. We carried out extensive qualitative and feasibility work with families prior to conducting the trial to ensure that their opinions and experiences informed both the intervention components and the outcome measures accordingly. Findings will be disseminated via the Public and Patient Involvement (PPI) group established in the context of the larger programme of research related to this trial. No further PPI involvement took place.

## Results

**Figure 1:**
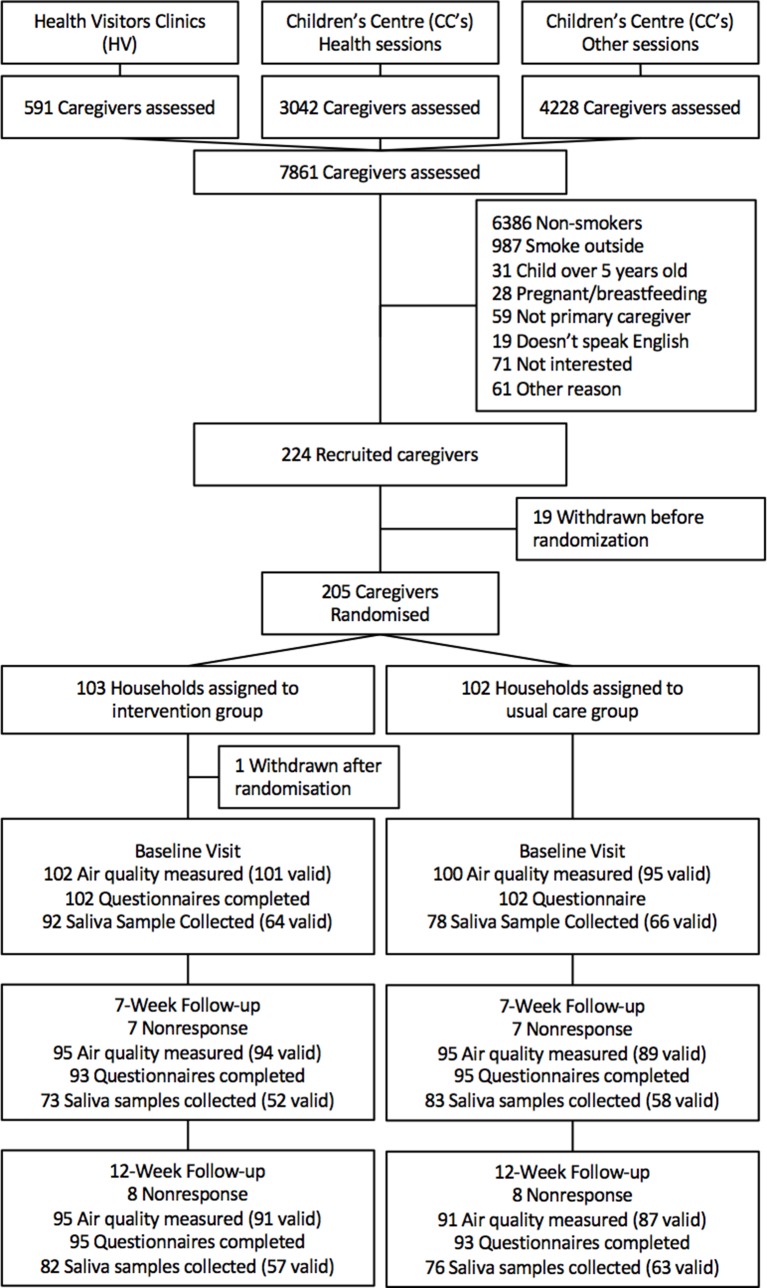
Approach, assessment for eligibility, randomisation and follow-up.

We assessed a total of 7861 caregivers, 1495 of whom were smokers with an interest in study participation. Of these, 297 fulfilled our eligibility criteria and 205 were randomised: 103 to intervention and 102 to usual care (figure 1), between October 2012 and September 2015. Baseline demographic and smoking-related data are shown in table 1. On average, participants’ multiple index of deprivation^25^ indicated high levels of social disadvantage (within the lowest quintile of the national range), and the majority lived in homes owned by the local authority. The number of cigarettes consumed inside the home per day was reported to be approximately 15, with the HSI indicating moderate levels of caregivers’ tobacco dependence. In both groups, the majority of caregivers declared not having a partner who cohabited with them all the time and had two children on average living in their household.

**Table 1:** Baseline characteristics of the study participants by treatment group^*^

	Intervention (n=103)	Usual care (n=102)
Socioeconomic status		
Multiple Deprivation Index rank^†^	6569.7 (5432.5)	6643.1 (5679.0)
Housing tenure		
Own/mortgaged	6 (6%)	9 (9%)
Private rent	48 (47%)	37 (36%)
Council/local authority	47 (46%)	53 (52%)
Other	2 (2%)	3 (3%)
Adults, n	1.5 (0.6)	1.6 (0.7)
Children, n	2.1 (1.2)	2.2 (1.2)
Age of children, years	3.6 (2.6)	3.3 (2.3)
Cigarettes smoked daily inside the home, n		
Mean (SD)	15.0 (11.0)	15.0 (11.0)
Median (IQR)	12.0 (8.0–20.0)	12.0 (7.0–20.0)
Caregiver Heaviness of Smoking Index^‡^	2.6 (1.5)	2.5 (1.6)
Seasonality at baseline appointment		
Warm	56 (54%)	53 (52%)
Cold	47 (46%)	49 (48%)
Ethnicity		
Other	8 (8%)	4 (4%)
White British	95 (92%)	98 (96%)
Partner cohabits all of the time		
Other	60 (58%)	58 (57%)
Yes	43 (42%)	44 (43%)
Age of caregiver, years	28.1 (6.2)	27.9 (6.6)
Air quality at home (PM_2.5_), µm/m^3§^		
Mean (SD)	54.6 (71.1)	46.5 (52.8)
Median (IQR)	33.4 (12.3–77.8)	30.5 (14.1–72.7)
Maximum PM_2.5_	437.5 (590.6)	401.7 (433.6)
Cotinine index child, ng/mL		
Mean (SD)	7.5 (8.1)	7.6 (8.0)
Median (IQR)	4.76 (6.21)	4.77 (7.48)

*Means and SD for most variables, unless otherwise stated. There was no statistically significant difference between groups in any of the variables listed.

†The Index of Multiple Deprivation rank goes from 1 (most deprived area) to 32 844 (least deprived area).

‡Heaviness of Smoking Index derives from Fagerström Test for Nicotine Dependence but uses only two questions (how soon after waking up do you smoke your first cigarette and how many cigarettes a day do you smoke). Scores range from 1 to 6, where higher scores are for higher dependency and lower scores are for lower dependency.

§Indoor air pollution concentration, 24 hours average PM_2.5_ (21), and in our case, 16–24 hours average PM_2.5_ is measured in µm/m^3^, which refers to milligrams of pollutant per cubic metre of air (in this case the pollutant is PM_2.5_).

**Figure 2:**
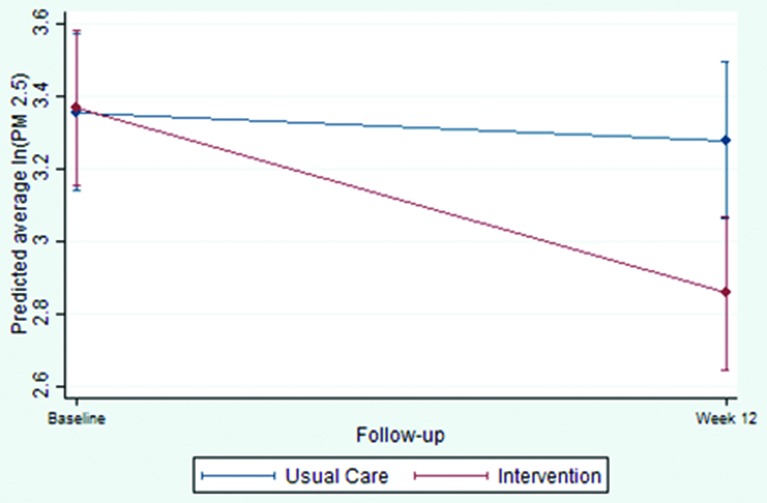
Predicted 16–24 hours average PM_2.5_ (log transformed) and 95% CIs by group and follow-up from the mixed model.

The primary analysis by multiple imputation showed a significant (p<0.001) reduction in 16–24 hours average PM_2.5_ at 12 weeks in the intervention group compared with the usual care group, adjusted for baseline and additionally for other prognostic factors (see Table 2, supplementary table1). Very similar results were obtained in the sensitivity analyses. The multiple imputation coefficient of −0.45 (p<0.001) denotes, when unlogged, a decrease of air pollution in the intervention group of 36.3%. Taking into account this reduction and the skewedness of the primary outcome, a participant in the median of the distribution, with a 16–24 hour average PM_2.5_ of 28.3 μg/m^3^ would have a concentration of 18.3 μg/m^3^ of PM_2.5_ by the end of week 12, which is below WHO-recommended safe levels of indoor 24 hours PM_2.5_ concentration.^23^ Figure 2 depicts predicted log-transformed 16–24 hours average PM_2.5_ from the mixed model for participants in the intervention and usual care groups. The model estimated a difference between groups at week 12 of −0.43 (95% CI −0.73 to −0.14), equating to a decrease of 35.2% (95% CI 12.7% to 51.9%).

10.1136/tobaccocontrol-2016-053279.supp1 Supplementary table 1

Analysis of secondary outcomes (see Table 3, supplementary table 2) showed that the proportion of time spent above 25 µg/m^3^ in the home and salivary cotinine from the index child, valid and comparable for 47.1% of the sample, had decreased in the intervention compared with the usual care group, although the latter was statistically significant only in the adjusted analysis (p=0.04). The number of cigarettes smoked in the home and HSI were both reduced significantly in the intervention compared with the usual care group both in basic and adjusted analysis. There was a threefold increase in the odds of making an attempt to quit during the 12 weeks of the study in the intervention group compared with usual care, whereas the proportion of those who had quit altogether was not significantly different between groups, and determination to quit was higher in the usual care group at 12 weeks. No important unintended harms resulted from the intervention in either group.

The vast majority of participants in the intervention group had received air quality feedback and behavioural support at baseline, week 7 and week 12; approximately two-thirds were using NRT products at week 12. Table 2 details the information related to adherence to the intervention and participants’ perception of importance of single intervention components received.

**Table 2:** Adherence to intervention components and importance ranking at week 12 for the intervention group (number and percentage in brackets)

	Baseline (%)	Week 7 (%)	Week 12 (%)
Intervention group study participants	102 (100)	95 (93)	95 (93)
Received valid air quality feedback	101 (99)	94 (92)	91 (89)
Received behavioural support	102 (100)	95 (93)	95 (93)
Used any NRT during the study^*^	–	73 (79)	64 (67)
Used inhalator	–	39 (42)	31 (33)
Used gum	–	36 (39)	24 (26)
Used lozenge	–	22 (24)	16 (17)
Used patch	–	15 (16)	13 (14)
Used quick mist	–	22 (24)	16 (17)
Used other	–	2 (2)	1 (1)
Ranking of intervention components			
Ranked air quality feedback first	–	–	65 (68)
Ranked behavioural support first	–	–	13 (14)
Ranked NRT first	–	–	17 (18)
Ranked air quality feedback second	–	–	21 (22)
Ranked behavioural support second	–	–	49 (52)
Ranked NRT second	–	–	24 (26)
Ranked air quality feedback third	–	–	10 (11)
Ranked behavioural support third	–	–	31 (33)
Ranked NRT third	–	–	53 (56)

*Considers the use of NRT after first advice from smoke-free homes advisors.

NRT, nicotine replacement therapy.

## Discussion

In this randomised controlled trial, a complex intervention combining personalised feedback on home air quality with behavioural and pharmacological support achieved significant behaviour change, improvements in indoor air quality and consequent reductions in exposure of young children to SHS as measured by salivary cotinine. Notably, these changes took place in predominantly highly disadvantaged single-parent families who, at the outset of the study, were not willing to consider quitting smoking. To our knowledge, this is the first trial to demonstrate effectiveness of this type of intervention across a comprehensive range of primary and secondary outcome measures, including home air quality, child salivary cotinine, smoking and quitting behaviour. It demonstrates that an intensive intervention can succeed in preventing harm to children from SHS even in the most difficult circumstances, with the potential to break the intergenerational cycle of tobacco addiction and smoking-related harm. Although we did not investigate measures relating to the potential clinical effectiveness of reduced secondhand smoke exposure in the home on respiratory and other smoking-related conditions, our findings could be of particular relevance, in view of discussions relating to smoke-free public housing^9^ and the circumstance that recent trials of minimal interventions to achieve reduced secondhand smoke exposure have shown to be unsuccessful.^26^

Although all of the families enrolled in our trial belonged to low socioeconomic strata, and almost all were of white British background, our findings are likely to be generalisable to families of other socioeconomic or ethnic backgrounds with the shared common feature of a caregiver who feels unable or unwilling to quit smoking. However, in view of previous findings indicating that barriers to creating SFHs are particularly great among disadvantaged families,^8^ our results are of particular relevance and may have underestimated the effect the intervention could have in less challenging environments. Our findings do not appear to have been adversely affected by the calibration error that generated inflated home air quality measurements at baseline and/or at week 7 for 29 participants, which was corrected for analysis of indoor air quality but resulted in the delivery of behavioural advice to these participants based on inflated readings. Although this may have affected motivation and behaviour change positively or negatively, depending on the context provided by previous measurements, our sensitivity analysis demonstrates that the error had little effect on key study outcomes.

In terms of the primary outcome (change in indoor air quality at 12 weeks), it is of note that particulate matter was sampled at various points using air monitors over a period of up to 24 hours. It is perceivable, particularly given the presence of the monitor inside the home, that participants could have adjusted their smoking behaviour to affect air quality readings. Although this is a possibility that we cannot rule out, it is important to consider that we collected a number of other outcome measures, including salivary cotinine from the index children, that are less likely to be affected by short-term behaviour change or other bias. Results from our analysis of salivary cotinine measures were also significant, underpinning the likelihood of our findings related to indoor air quality being reliable. PM_2.5_ is an indirect but well-established measure of secondhand smoke and has been used extensively to assess secondhand smoke levels in homes, and elsewhere.^27^ It can be affected by non-tobacco sources, including cooking, heating and environmental exposures,^27^ but because our study was randomised and there is no reason to suspect that any change over time from other sources would affect the intervention group differentially from control, it is unlikely that this has affected our results.

The results of this study build on the Reducing Families' Exposure to Secondhand Smoke in the Home (REFRESH) feasibility study, which only showed evidence of effectiveness within and not between treatment groups,^17^ suggesting that the more intensive and longer intervention period is important to support the initiation and maintenance of SFH to protect children from exposure to SHS. The reduction in levels of SHS exposure found in our study of caregivers who were unable or unwilling to quit smoking is similar to that reported in the American KISS Trial,^28^ which, however, included caregivers who had recently quit or tried to do so. This could be interpreted as evidence that our intervention was particularly effective because our population may have been less amenable to smoking-related behaviour change. Our findings also demonstrate intervention effectiveness across a range of further smoking-related measures, such as increases in determination to quit, and actual quit attempts, which predict further quit attempts in the future.^29^

This is in contrast to a US trial assessing a multicomponent intervention including motivational interviewing, where effectiveness was found for improvement of air quality in the home only at 12-month follow-up but not before and not at all for salivary cotinine.^30^ It is possible that our intervention was more effective, because it was delivered in private family homes rather than largely in a public school environment, included NRT and personalised advice from trained smoke-free homes advisors to make the family home smokefree, thus being particularly tailored to individual need. In contrast also to a recently published RCT of a complex intervention very similar to the one used in our study, conducted with 205 families in Armenia^31^ using children’s hair cotinine as primary outcome measure, our findings of changes in salivary cotinine were significant in the adjusted analysis, although similar challenges in collecting samples for cotinine analysis from index children were experienced. There was evidence in our study that determination to quit smoking was greater in participants who had received the low-intensity ‘usual care’ intervention, which could be interpreted in various ways. For example, it could indicate satisfaction of complex intervention participants with their achievements and improved home air quality for their children, removing the perceived ‘necessity’ to stop smoking completely. However, the significantly higher number of quit attempts during the study in the intervention group, measured at various time points, contradicts this theory.

Overall, our intervention specifically contributes to the growing evidence base of effective multicomponent interventions that result in a reduction in PM_2.5_, which helps to protect children from exposure to SHS in the home.^16^ In addition, it helps to provide important information on intervention components and modalities that can be used to inform future meta-analyses/evidence syntheses given that, to date, systematic review evidence^13 14 16^ has not been able to recommend one specific intervention over another. It is important to notice that, as with other studies,^16^ our intervention did not eliminate SHS exposure in the participating homes. The need to develop and test complex interventions that have the potential to do so remains apparent.

The high retention of participants in our study can be viewed as a strength and as an indicator of high motivation among smokers to protect their children from SHS exposure, denoting the potential of widespread uptake of interventions to support this aim. The combination of a financial incentive on completion of data collection, free NRT, frequent communication with SFHAs and flexibility to accommodate participants’ needs in terms of appointments is also likely to have contributed to the high retention.

An important consideration refers to the cost-effectiveness of the complex intervention tested here. An economic analysis is currently ongoing and will be made available elsewhere. Further research to refine our intervention, adapting it for widespread and sustainable application and taking into account participants’ perceived importance of intervention components is required. Rankings of importance obtained from our intervention participants indicate the central role of personalised air quality feedback (ranked as the single most important intervention component by most participants); further exploration of this and implications for future service development and implementation is warranted. Furthermore, research related to clinical impacts of reduction in SHS exposure is also required.

**What this paper adds**

- Children’s exposure to secondhand tobacco smoke (SHS) is extremely harmful to health and occurs mainly in the home. Behavioural and pharmacological interventions can improve air quality in the homes of smoking caregivers motivated to quit smoking, thus reducing children’s harmful exposure to secondhand smoke in the home.
- A complex intervention including behavioural and pharmacological support and air quality feedback was effective in reducing children’s exposure to SHS in the homes of caregivers who were not motivated to quit in deprived communities and improved a range of smoking-related outcome measures, including saliva cotinine, daily cigarette consumption and quit attempts. It highlights the potential of such interventions to break the intergenerational cycle of tobacco-related harm even under the most difficult circumstances.

10.1136/tobaccocontrol-2016-053279.supp2 Supplementary table 2
