# Supplementary material for: A randomised controlled trial of a complex intervention to reduce children’s exposure to secondhand smoke in the home
Source: Tob Control. 2017 Apr 21;27(2):155–62. doi: 10.1136/tobaccocontrol-2016-053279 (PMC5870442; doi:10.1136/tobaccocontrol-2016-053279)
Supplement: Supplementary file 1 [file tobaccocontrol-2016-053279supp003.docx]

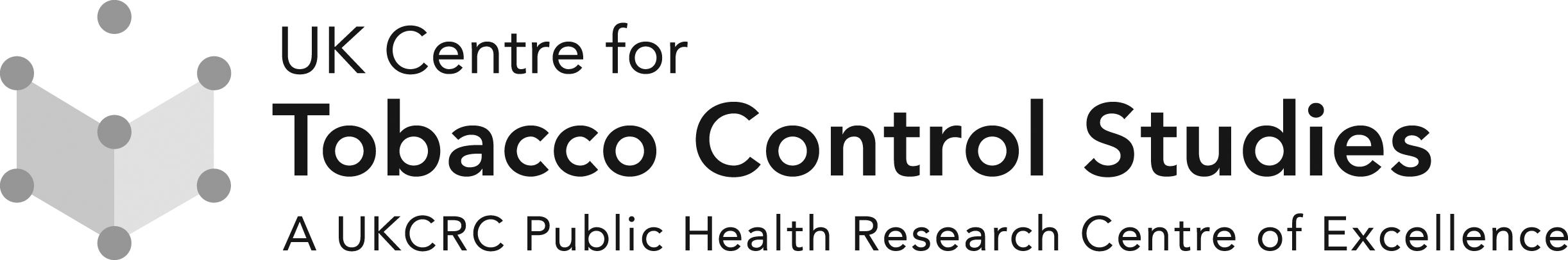

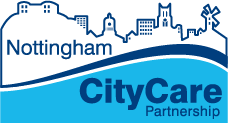

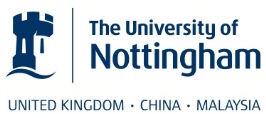

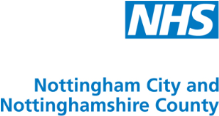


## **SFH Fact Sheet**

## (Final version 1.0: 14/08/2012)

**Title of Study:** Assessing an intervention to reduce children’s exposure to secondhand smoke at home

**Name of Researchers*:*** Dr Elena Ratschen, Dr Laura Jones and Mrs Rebecca Thorley

**Making your home smoke-free: the facts**

**What is a smoke-free home?**

A home where nobody smokes inside, not even at the window or door, and where this rule is **NEVER** broken.

**What is secondhand smoke (SHS)?**

SHS is made up of two different types of smoke:

1. Mainstream smoke: Smoke that comes from the burning end of a cigarette.

2**.** Side-stream smoke: Smoke that is exhaled by smokers.

**By smoking and allowing others to smoke in your home, you are increasing your child’s risk of:**

- Sudden unexpected death in infancy

- Lower respiratory tract infections

- Asthma & wheeze symptoms

- Middle ear disease

- Bacterial meningitis

**Smoking with the doors and/or windows open and using air fresheners:**

makes no difference to your child’s level of exposure.

**85% of secondhand smoke is invisible:**

you might not be able to see it or smell it, but it’s still there and is harming your children.

**Making your home smoke-free: top tips**

**Not ready to quit?**

That’s fine. Remember that we’re not asking you

to quit, we’re asking you to take smoking completely

outside of the house to protect your family from

the harms of SHS exposure**.**

**Nicotine replacement therapy (NRT):**

Using the NRT (e.g. patches) that you received from

your smoke-free homes advisor will help take away

your cigarette cravings when you can’t

safely get outside to smoke.

**How do I raise the issues with others?**

We know that this can be really difficult:

- You could start by saying that you’re taking part

in this study.

**Then you can say “I’ve been thinking about maybe**

trying to get everyone to smoke outside, so that

the kids don’t breathe it in and so that my house

doesn’t smell” and see what they have to say,

you might just be surprised.

- Be confident in yourself.

**It’s cold outside!**

Keep a coat by the door that you can put on to keep you warm when you go outside to smoke.
