# Supplementary material for: A randomised controlled trial of a complex intervention to reduce children’s exposure to secondhand smoke in the home
Source: Tob Control. 2017 Apr 21;27(2):155–62. doi: 10.1136/tobaccocontrol-2016-053279 (PMC5870442; doi:10.1136/tobaccocontrol-2016-053279)
Supplement: Supplementary table 1 [file tobaccocontrol-2016-053279supp001.docx]

*Supplementary Table 1: Primary and Sensitivity Analysis: Changes between Baseline and Week Twelve for 16-to-24-hour Log Transformed Average PM2.5 (average PM2.5 in levels in squared brackets)*

|  | Intervention | | Usual Care | | Mean Difference | | | |
| --- | --- | --- | --- | --- | --- | --- | --- | --- |
|  | Baseline  (SD) | Week 12  (SD) | Baseline  (SD) | Week 12  (SD) | Basic^*^  (95% CI) | P Value | Adjusted^†^  (95% CI) | P Value |
| **Primary Outcome Analysis** | | | | | | | | |
| Multiple Imputation^‡^ | 3^.^4 (1^.^1)  [54^.^2] | 2^.^9 (1^.^0)  [30^.^7] | 3^.^4 (1^.^1)  [48^.^7] | 3^.^3 (1^.^2)  [47^.^6] | -0^.^45  (-0^.^75 to -0^.^16) | 0^.^003 | -0^.^45  (-0^.^75 to -0^.^16) | 0^.^003 |
| No. households | 102 | 102 | 102 | 102 | 204 |  | 204 |  |
| **Sensitivity Analysis** | | | | | | | | |
| Complete Case | 3^.^4 (1^.^1)  [54^.^6] | 2^.^8 (1^.^0)  [28^.^9] | 3^.^3 (1^.^0)  [46^.^5] | 3^.^3 (1^.^1)  [49^.^8] | -0^.^39  (-0^.^66 to -0^.^13) | 0^.^004 | -0^.^39  (-0^.^66 to -0^.^13) | 0^.^004 |
| No. households | 101 | 91 | 95 | 87 | 172 |  | 172 |  |
|  |  |  |  |  |  |  |  |  |
| Last Observation Carried Forward | 3^.^4 (1^.^1)  [54^.^6] | 2^.^9 (1^.^0)  [33^.^1] | 3^.^3 (1^.^0)  [46^.^5] | 3^.^3 (1^.^1)  [49^.^0] | -0^.^32  (-0^.^56 to -0^.^08) | 0^.^01 | -0^.^32  (-0^.^56 to -0^.^08) | 0^.^01 |
| No. households | 101 | 102 | 95 | 100 | 196 |  | 196 |  |
|  |  |  |  |  |  |  |  |  |
| Mixed Model^§^ | 3^.^4 (1^.^1)  [54^.^6] | 2^.^8 (1^.^0)  [28^.^9] | 3^.^3 (1^.^0)  [46^.^5] | 3^.^3 (1^.^1)  [49^.^8] | -0^.^43  (-0^.^73 to -0^.^14) | 0^.^004 | -0^.^30  (-0^.^60 to 0^.^00) | 0^.^05 |
| No. households | 101 | 91 | 95 | 87 | 202 |  | 202 |  |
|  |  |  |  |  |  |  |  |  |
| Excluding households with affected data due to calibration error^‖^ | 3^.^4 (0^.^9)  [55^.^9] | 3^.^0 (0^.^9)  [33^.^4] | 3^.^4 (1^.^0)  [51^.^5] | 3^.^3 (1^.^0)  [47^.^9] | -0^.^40  (-0^.^72 to -0^.^09) | 0^.^01 | -0^.^40  (-0^.^71 to -0^.^09) | 0^.^01 |
| No. households | 86 | 86 | 91 | 91 | 177 |  | 177 |  |
|  |  |  |  |  |  |  |  |  |
| Average PM_2.5_ paired for each participating household^‖^ | 3^.^4 (1^.^0)  [53^.^6] | 2^.^8 (1^.^0)  [30^.^7] | 3^.^3 (1^.^0)  [48^.^7] | 3^.^2 (1^.^0)  [42^.^9] | -0^.^40  (-0^.^72 to -0^.^09) | 0^.^01 | -0^.^41  (-0^.^72 to -0^.^09) | 0^.^01 |
| Average PM_2.5_ paired for all participating households^‖^ | 3^.^5 (1^.^0)  [67^.^9] | 3^.^0 (1^.^0)  [37^.^2] | 3^.^5 (1^.^0)  [60^.^7] | 3^.^4 (2^.^0)  [57^.^8] | -0^.^38  (-0^.^80 to 0^.^05) | 0^.^08 | -0^.^37  (-0^.^81 to 0^.^06) | 0^.^09 |
| No. households | 102 | 102 | 102 | 102 | 204 |  | 204 |  |

^*^ Basic refers to adjusted only for baseline log transformed average PM_2_^.^_5_.

^†^ Adjusted for baseline log transformed average PM_2_^.^_5_, as well as for season at week twelve, deprivation index and having a partner who smokes.

^‡^ Imputing five datasets

^§^ In the mixed method model the effectiveness measure is the coefficient associated to the interaction between Intervention Group and Week Twelve Visit.

^‖^ Using multiple imputation model (imputing five datasets).
