# Supplementary material for: A randomised controlled trial of a complex intervention to reduce children’s exposure to secondhand smoke in the home
Source: Tob Control. 2017 Apr 21;27(2):155–62. doi: 10.1136/tobaccocontrol-2016-053279 (PMC5870442; doi:10.1136/tobaccocontrol-2016-053279)
Supplement: Supplementary table 2 [file tobaccocontrol-2016-053279supp002.docx]

*Supplementary Table 2: Secondary Outcome Measures: air quality, smoking & quitting behaviour^*^ (natural values of transformed variables in squared brackets)*

|  | **Intervention** | | **Usual Care** | | **Mean Difference (95% CI)** | | | |
| --- | --- | --- | --- | --- | --- | --- | --- | --- |
|  | Baseline  (SD) | Week 12  (SD) | Baseline  (SD) | Week 12  (SD) | Basic^†^  (95% CI) | P Value | Adjusted^‡^  (95% CI) | P Value |
| No. Households | 101 | 91 | 95 | 87 | 172 |  | 172 |  |
| Ln(max. PM_2.5_^§^) | 5^.^4 (1^.^2)  [441^.^6] | 4^.^9 (1^.^4)  [463^.^1] | 5^.^4 (1^.^1)  [401^.^7] | 5^.^3 (1^.^2)  [359^.^3] | -0^.^31  (-0^.^67 to 0^.^04) | 0^.^09 | -0^.^32  (-0^.^67 to 0^.^04) | 0^.^08 |
| Proportion of time spent over 25 µm/m^3§^ | 0^.^4 (0^.^3) | 0^.^2 (0^.^3) | 0^.^3 (0^.^3) | 0^.^3 (0^.^3) | -0^.^12  (-0^.^20 to -0^.^05) | 0^.^001 | -0^.^12  (-0^.^19 to -0^.^05) | 0^.^001 |
|  |  |  |  |  |  |  |  |  |
| No. Households | 64 | 57 | 66 | 63 | 96 |  | 96 |  |
| Ln(Cotinine levels) | 1^.^6 (0^.^9)  [7^.^5] | 1^.^4 (0^.^9)  [6^.^5] | 1^.^6 (0^.^9)  [7^.^6] | 1^.^6 (1^.^0)  [8^.^1] | -0^.^26  (-0^.^58 to 0^.^05) | 0^.^10 | -0^.^32  (-0^.^64 to 0^.^01) | 0^.^04 |
|  |  |  |  |  |  |  |  |  |
| No. Households | 102 | 52 | 102 | 76 | 128 |  | 128 |  |
| Ln(No. of cigarettes smoked daily at home) | 2^.^4 (0^.^8)  [15^.^1] | 1^.^6 (1^.^0)  [7^.^3] | 2^.^4 (0^.^8)  [15^.^0] | 2^.^3 (1^.^0)  [13^.^4] | -0^.^77  (-1^.^08 to -0^.^47) | 0^.^000 | -0^.^77  (-1^.^07 to -0^.^46) | 0^.^000 |
|  |  |  |  |  |  |  |  |  |
| No. Households | 102 | 87 | 102 | 88 | 175 |  | 175 |  |
| Heaviness of Smoking Index | 2^.^6 (1^.^5) | 1^.^8 (1^.^5) | 2^.^5 (1^.^6) | 2^.^2 (1^.^6) | -0^.^50  (-0^.^80 to -0^.^19) | 0^.^001 | -0^.^50  (-0^.^81 to -0^.^20) | 0^.^001 |
|  |  |  |  |  |  |  |  |  |
|  | **Intervention** | | **Usual Care** | | **OR (95% CI)** | | | |
|  | Baseline | Week 12 | Baseline | Week 12 | Basic^†^ | P Value | Adjusted^‡^ | P Value |
| No. Households | 102 | 95 | 102 | 93 | 188 |  | 188 |  |
| Seriously planning to quit smoking | 77  (75^.^5%) | 70  (73^.^7%) | 81  (79^.^4%) | 74  (79.6%) | 0^.^72  (0^.^36 to 1^.^42) | 0^.^34 | 0^.^72  (0^.^36 to1^.^42) | 0^.^36 |
|  |  |  |  |  |  |  |  |  |
| No. Households | 93 | 95 | 95 | 93 | 188 |  | 188 |  |
| Has attempted to quit during the study^‖^ | 19  (20^.^4%) | 20  (21^.^1%) | 5  (5^.^3%) | 7  (7^.^5%) | 3^.^28  (1^.^31 to 8^.^20) | 0^.^01 | 3^.^25  (1^.^30 to 8^.^11) | 0^.^01 |
|  |  |  |  |  |  |  |  |  |
| No. Households | 93 | 95 | 95 | 93 | 188 |  | 188 |  |
| Has quit smoking during the study^‖^ | 5  (5^.^4%) | 8  (8^.^4%) | 3  (3^.^2%) | 4  (4^.^3%) | 2^.^05  (0^.^59 to 7^.^07) | 0^.^26 | 2^.^04  (0^.^56 to 7^.^42) | 0^.^28 |

^*^ Mean and SD reported for baseline and week twelve mean differences and number and percentage –no. (%) –reported for baseline odds ratios

^†^ Basic refers to adjusted by baseline for mean differences and just group difference for odds ratio

^‡^ Adjusted refers to basic model adjusted by season at week twelve, deprivation index and having a partner who smokes.

^§^ Computed for those participants with 16 to 24 hours of PM_2.5_ measurements.

^‖^ Measured at week seven and week twelve, hence comparison is between these two times.
